# How to Establish a Dental Practice

**Published:** 1898-01

**Authors:** N. S. Hoff


AMERICAN JOURNAL

—OF—

DENTAL SCIENCE.

VOL. XXXI. BALTIMORE, JANUARY, 1898. No. 9.

ARTICLE I.

### HOW TO ESTABLISH A DENTAL PRACTICE.

BY N. S. HOFF, D. D. S.

There must be a personality. I am aware of the fact that many practices are established that are in certain directions and in some measures successful, and which seem to be entirely impersonal. Patients visit the New York, or Boston, or Battle Creek dental parlors and put themselves under the care of no one in particular, but like chickens hatched in incubators, reared by a wooden box with cloth skirts and marmed by an oil lamp—a poor substitute for a vigilant, fat and well-fed mother hen. But I do not claim to know anything about hatching out dental practices on the incubator plan. I wasn't brought up that way, and of course I have little sympathy with this kind of practice building. In the kind of practice that I think we all crave, the personality, while not everything, is so important that I give it the first place.

We cannot regenerate men physically as we can morally. But I believe where incurable physical deformities exist much can be done, with a proper appreciation of the requirements necessary for the adoption of one to his environment. A man that is physically ill-formed may not be able to modify his conditions to make himself acceptable to everybody, and yet many an ill-natured person by persistent effort, thought and care taking has been able to so modify his unattractiveness as to make himself entirely tolerable. I believe that all persons having such disabilities should be advised of their disadvantages before entering the profession. But when one is qualified and prepared for practice these natural disabilities should not deter him from making a heroic effort to so conceal them with other attractive qualities as to have himself recognized in spite of his misfortune. Neither a blind man nor an armless man could practice dentistry successfully. There are many persons, however, trying to practice dentistry with defective eyes, and, unconsciously perhaps, are not availing themselves of the aids a good oculist could give them. There are many persons with crippled fingers, stiff joints and lack of acute tactile sense of the fingers, all of which stand in the way of successful practice. The skilful surgeon, a course of massage, or persistent attention to the care of the skin by systemic and local treatment would soon enable a bungling operator to appreciate the difference in the size of a lead pencil and nerve broach.

It is important to take into account temporary disqualifications, conditions of neglect in clothing, toilet, or the presence of disease or its results. These may be and usually are offensive, and will keep patient and dentist out of harmony with each other. No delicate and refined lady wants to have for her dentist a man who smokes, neither will such a one long tolerate a person whose breath suggests the garbage pail from his indulgence in malt liquors, fragant cheese and odoriferous relishes. Catarrhal trouble without proper care is bound to be offensive. If ther is indigestion the conditions certainly demand peremptory correction. Eruptions in exposed parts of the body and other offensive conditions are to be scrupulously avoided. Strict attention to personal habits of cleanliness, combined with clean and well selected clothing and ornaments, are of the greatest moment, and scrupulous cleanliness, both internally and externally, are necessary and to be constantly striven for and kept in mind. It is above all necessary that the dentist should be above reproach or suspicion morally. It is not for me to formulate or quote a moral code for any one. The dentist should be an honorable gentleman, and it will not be any drawback to be known and recognized as an active and energetic member in good standing of some religious organization. Of course I would not have any one infer that a church membership is to be valued as a business aid only. A person who would make use of any sacred organization for his own selfish purposes deserves the severest contempt of every right-minded person. But it is legitimate for a person to make use of the church, and any or all other organizations which he can command, to exalt and ennoble his own character. And no personality which is not in some way influenced by Christian religion or morals is fully equipped to make the most out of his opportunity to serve a large and appreciative support.

Again, an educated person will have decided advantage over one whose entire store of knowledge is confined to his profession, or is limited and characterless. Patients respect culture in their dentist as in their preacher, doctor or lawyer. The broader a man's knowledge the wider will be his influence professionally. What are the essentials? Every dentist who hopes to get and retain the confidence and support of his patients must be well qualified to perform all ordinary service in a good and substantial manner. This implies a thorough course of training in all the technical functions of dentistry, as well as such theoretical study as shall be necessary to qualify him to correctly diagnose diseased conditions, and to apply proper and adequate remedies for relief. To obtain this degree of proficiency he must set a high mark for attainment; one that cannot be reached by a three years' or five years' course of study in any dental educational institution, but which shall only be reached after years of careful and pains-taking effort to do the best possible for every case under-taken. Simple as well as difficult cases should be subjected to the closest scrutiny, that nothing of value or interest in any case may be overlooked. The early establishment of a systematic method in making diagnoses, and the treatment of every case by principles rather than without interest or mechanically, is of the utmost importance. No dentist who performs his work in a perfunctory manner will respect or love his work, and his patients will know of it before he fully appreciates it himself. The man who cobbles a shoe takes pride in his work in proportion to the skill and thought he puts into it. The scientist, diplomat or statesman takes pride in his work only so far as he has put his best into it, and the dentist who will put all the brains he can into his work will soon find his heart enlisted, and it will be marvelous indeed to have such a combination defeated. We always find that the man who gets thus interested in his own work and becomes attached to his calling, and finds himself appreciated by the community in which he lives, begins to think of his professional brethren, and he wants to know what they are thinking and how they get along with their work, and he subscribes for the dental magazines and begins to read, and then he wants to see these men who write and talk in society meetings. He wants to ask them some questions that he finds difficult to adjust, and so he joins his local, state or national dental society, and he attends the meetings as religiously as he does his church prayer-meeting, and he feels in duty bound to take his part in the dental meeting just as much as in his church society, and thus he gets interested in his profession and his patients know it without any announcement on his part through the press, and are glad of it and proud of their dentist, not because he announces in all the local papers that he is such a big man away from home, that he has been announced by the dentists in national assemblage to tell them how he does it, but because they are glad that he is broad and liberal enough to take an interest in his calling, and is willing to sacrifice his time and money to get on in the calling to which he is devoting his life.

A very wealthy and intelligent man, a warm, personal friend of mine, said to me, when I was a young professional man, that he would like to come to me professionally, and send his family to me, but he said he had always gone to another dentist, and had paid him large fees willingly, because he knew that he gave him his best services, and further that he believed that he was a thoroughly professional man, and would use whatever money he paid him for the advancement of his profession. To me this statement was an eye-opener, and I have many times wondered why all people did not hold the same view. I really believe more of our patients have something of this idea than we think, though they may not be conscious of it or be able to formulate it into any well defined theory for action.

If we expect to attract intelligent and discriminating people professionally we must be the embodiment of the highest ideas of the professional. We must actually live the life, not theoretically nor ostentatiously, but honestly and conscientiously.

There must also be the faculty or ability to conduct professional business on legitimate and conscientious business principles. The comment is frequently made that professional people are notabty deficient in business methods or habits. Few professional men accumulate large properties, although they may do considerable business and at good profit. Professional men ought to lay by a portion of their earnings, and begin early to do so, and keep everlastingly at it, and employ the safest means available for caring for these accumulations until such time as they are of sufficient amount to purchase productive real estate or safe mortgages.

But there is another way in which dentists, doctors and lawyers are frequently accused of being bad business men. This is in not meeting their obligations promptly. There are of course rascals in the dental as well as in other callings, but all dentists who do not pay their debts promptly are not rascals, at least not intentionally. Many professional men are tempted or educated into this lax habit of not paying bills promptly by their patients. Doctors are probably the worst paid of all professional men, perhaps lawyers next, and, owing to their close relationship, dentists' bills are put off until the landlord, the grocer and baker are all paid, and if anything is left the dentist gets it, and after he is paid the doctor may get a portion. A dentist in full practice realizes that he is making a profit, and naturally thinks he has the right to use it for such necessities and luxuries as he may desire, and consequently spends or contracts to spend much of his money before it is collected. Or if he is not so reckless, he reasons that when others owe him he has a right to hold off payment of his debts until some convenient time. This is a kind of logical justice that has no support in the moral code. Nevertheless, it operates largely, and with men who would not like to be called selfish or dishonorable. The moral is that to have the confidence of the business community a dentist should pay his debts as promptly as it is possible, and never to spend his earnings until in hand. To do this he will need to give some thought to the manner of making his collections regularly and systematically, and without offense to his patrons. This brings up the whole subject of keeping accounts and records, and the best methods for regulating fees and of making collections which I have no time to discuss here; but I do want to say that the business part of conducting a dental practice is exceedingly important, and should not be carelessly nor thoughtlessly managed. I have heard it said of dentists that their fees are often based not on the character and amount of the service rendered so much as on the ability of the patient to pay or the pressing needs of the dentist. I once had a dentist relate to me how he worked the fee he first planned to charge to more than double, simply because he had a willing patron and his wife insisted on his buying a handsome velvet cloak for their daughter. Such business ideas are not only selfish, but contemptible and dishonorable, and no man who practices them will permanently succeed, nor should he. Such habits will unquestionably demoralize business capacities and wreck professional life, because the aim will eventually be not how well can the work be done, but how quickly and at the least possible outlay of effort, mental and physical. Its result is to be found in the miserable attempts we see all around us, to get the attention of the unwary by resorting to all sorts of schemes to get the public to take notice of the great and only Dr. Quack, who extracts teeth free, without pain, from 9 to 12 o'clock on Saturdays, etc. Does any intelligent, honest or well-bred and self-respecting man want to have such a name or notoriety? So any one seeking such notoriety a more commendable method, and one not likely to injure innocent and unsuspecting persons, is to get together a menagerie of albino children, bearded women, double-headed calves, big snakes, etc., and erect a tent on some vacant lot, and with a hand organ to attract the crowd he can gratify his ambition of being seen and heard.

The vulgar display of our profession is not to be commended. It is not a good business idea for a young man, when he decides on a location, to embarrass his future business success with an extravagant outlay for furniture, equipment or excessive office rent, which he has no immediate nor remote probability of being able to pay. I would not, on the other hand, advise a man to outfit so meanly that refined persons would hesitate to approach his premises, or that he would be handicapped in his work by inferior or inadequate accessories. But no man is justified in making what exceeds the bounds of good judgment in assuming financial obligations which may cause him to lose his professional and business integrity. The chances of recovering ground once lost in this way are few, and it is better far that time should be sacrificed than honor or professional standing in the community and among the professional brethren.—*Dental Register.*

